# Characteristics of non-diabetic foot ulcers in Western Sydney, Australia

**DOI:** 10.1186/s13047-016-0137-6

**Published:** 2016-02-11

**Authors:** Norafizah Haji Zaine, Kerry Hitos, Mauro Vicaretti, John P. Fletcher, Lindy Begg, Joshua Burns

**Affiliations:** Arthritis and Musculoskeletal Research Group, Faculty of Health Sciences, The University of Sydney, Sydney, NSW Australia; Foot Wound Clinic, Department of Surgery, The University of Sydney, Westmead Hospital, Sydney, NSW Australia; Westmead Research Centre for the Evaluation of Surgical Outcomes, Department of Surgery, The University of Sydney, Sydney, NSW Australia; Podiatry Unit, Raja Isteri Pengiran Anak Saleha Hospital, Bandar Seri Begawan, BA1710 Brunei Darussalam

**Keywords:** Diabetic foot ulcers, Non-diabetic foot ulcers, Peripheral neuropathy, Ischaemia, Socioeconomic status

## Abstract

**Background:**

There are few studies investigating the characteristics, risk factors and socioeconomic status of patients with non-diabetic foot ulcers. The aim of this study was to explore the characteristics of non-diabetic foot ulcers in a large tertiary referral outpatient hospital setting in Western Sydney, Australia.

**Methods:**

From 2011 to 2013, data from 202 patients with non-diabetic foot ulcers during their initial visit were retrospectively extracted for analysis from Westmead Hospital’s Foot Wound Clinic Registry. Data including demographics, socioeconomic status and foot ulcer characteristics were recorded on a standardised data collection form.

**Results:**

Demographics and physical characteristics were: 54 % male, median age 78 years [interquartile range (IQR): 64–87], median body mass index (BMI) of 23.8 kg/m^2^ (IQR: 20–26.9), 35 % had loss of protective sensation and the median postcode score for socioeconomic status was 996 (IQR: 935–1034). Foot ulcer characteristics were: median cross-sectional area of 1.2 cm^2^ (IQR: 0.3–5.0), 30.5 % plantar and 27 % dorsal, 22.1 % University of Texas (UT) Wound Classification for Diabetic Foot Ulcers Grade of 1C-3C (with ischaemia).

**Conclusions:**

Unlike diabetic foot ulcers, non-diabetic foot ulcers largely affected older males *and* females. In accordance with diabetic foot ulcer characteristics, socioeconomic status was not related to non-diabetic foot ulcers in Western Sydney. Based on the findings of this study the epidemiological pattern of non-diabetic foot ulceration and its pathogenesis requires further investigation.

## Background

It is estimated that as many as 300,000 Australians have chronic wounds requiring management [1]. Wounds that do not heal within three months are often considered chronic [2]. Chronic and non-healing ulcers account for 69–77 % of all wound types [3]. Foot ulcers are commonly associated with diabetes and can be a major burden to patients and the health care system, especially those that recur or do not heal [4]. The two common types of foot ulcers are neuropathic and ischaemic followed by decubitus and malignant. These wounds often contain bacterial biofilms that can lead to chronic infections [5]. Foot ulcers also commonly occur in people without diabetes [4]. As with diabetic foot ulcers, these foot ulcers may develop due to overlapping factors including neuropathy, peripheral arterial disease, pressure overload, trauma and foot conditions such as fissures and callosities [6]. However, evidence concerning non-diabetic foot ulcer characteristics is scarce.

Whilst there are numerous studies investigating multiple high risk factors and foot ulcers in patients with diabetes [7, 8] studies on other at-risk populations are limited. Other chronic disease populations such as chronic kidney disease, cancer and cardiovascular disease have comparably high risk factors (such as hypertension and hyperlipidaemia) and foot ulcers to diabetes [9, 10]. There is a paucity of information on the characteristics and risk factors for foot ulcerations in a non-diabetic population in the Australian health care setting. In the largest database of foot ulcers in Australia [11], Lazzarini et al. examined the characteristics of ambulatory patients with a foot ulcer across 13 Health and Hospital Services and reported that of 2,034 people presenting with a foot ulcer, 15 % did not have a history of diabetes. One cross-sectional audit of health care professionals involved in the care of foot ulcers in the UK described 132 non-diabetic foot ulcers occurring in 54 % (*n* = 71) females and 46 % (*n* = 61) males [12]. They also showed that the ulcers were commonly located on the digits (*n* = 68, 52 %) followed by the heel (*n* = 33, 25 %), plantar surface (*n* = 16, 12 %) and dorsal aspect of the foot (*n* = 14, 11 %). Other studies have pooled leg and diabetic foot ulcers which makes it difficult to isolate non-diabetic foot ulcer characteristics [13, 14]. A similar retrospective study conducted in Western Sydney, Australia investigated the classification, characteristics, location of diabetic foot ulcers and the patients’ socioeconomic status. However, this study was on a diabetic population and data was extracted for a period of 1 year (2011) only [15].

There have been no studies exploring non-diabetic foot ulcers in the large Australian catchment of Western Sydney. It is unclear if the characteristics, risk factors and socioeconomic status of patients with diabetic and non-diabetic foot ulcers are similar. The aim of this study was to evaluate the characteristics of non-diabetic foot ulcers in a large tertiary referral outpatient hospital setting in Western Sydney, Australia. The secondary aim was to discuss foot ulcer commonalities and differences between this non-diabetic sample and a previously studied diabetic cohort [15].

## Methods

Ethical approval was granted by the Research Ethics Committees at the Western Sydney Local Health District and The University of Sydney. The study population was defined as the total number of patients without diabetes with foot ulcers at initial visit attending the outpatient Foot Wound Clinic at Westmead Hospital from January 2011 to December 2013. The Foot Wound Clinic is an interdisciplinary public health service for patients with foot ulcers (diabetic and non-diabetic), which is attended concurrently by podiatrists, vascular consultants and registrars, wound care consultants, vascular clinical nurse consultants and a clinic nurse. Infectious disease consultants are also available upon request. A foot ulcer was commonly defined as a full-thickness wound located distal to the ankle (level of malleoli) [16].

All data were captured in Westmead Hospital’s Foot Wound Clinic Registry. Data were extracted on a standardised data collection form. For inconsistencies such as ulcer size, location, classification and offloading modalities, clarification was sought from the treating clinician verbally or from the patient medical record. Patients with diabetes or without foot ulcers were excluded from the study. Background data included patient characteristics such as demographical details, socioeconomic status, marital status, country of birth and English language status (defined as patients who were English and non English speaking). Co-morbidities such as peripheral neuropathy, hyperlipidaemia, retinopathy, history of ulceration (healed) and/or amputation, angina/infarct, nephropathy, renal failure, claudication, cerebrovascular accident and transient ischaemic attack were recorded based on patient medical records, clinician referral letters and assessed.

Loss of protective sensation was diagnosed by a Podiatrist using a neurothesiometer, 128Hz tuning fork or 10 g monofilament according to a standardised protocol [17]. Investigations of foot ulcer related factors (such as peripheral arterial disease and ulcer infection), referrals to other health professionals, treatments (such as pressure offloading) and hospitalisation and/or requiring vascular or surgical interventions were also documented. Peripheral arterial disease (PAD) was assessed and diagnosed by measuring toe pressures using a photoplethysmography (Hadeco Smartdop 30 EX Vascular Ultrasound Doppler). A toe pressure of <30 mmHg indicates PAD and poor healing [18]. However toe pressures were excluded from further analysis due to missing data.

The socioeconomic status of each patient was based on the Australian Bureau of Statistics (ABS) residential postcode method for the general Australian population (mean index = 1000) [19]. The Index of Relative Socioeconomic Disadvantage (IRSD) is used by the ABS as a general socioeconomic index to summarise a range of information about the economic and social conditions of people and households within an area. A low score indicates relatively greater disadvantage whereas a high score indicates a relative advantage [20]. A score of less than 1000 indicates that the area is more disadvantaged than the average area at the Statistical Area Level 1 (SA1). SA1 is the smallest geographical unit at which the SEIFA (Socio-Economic Indexes For Areas) indexes are calculated [21].

Validated diabetic grading systems were used in the absence of validated non-diabetic foot ulcers measures. These were: information on osteomyelitis, foot ulcer PEDIS grades of infection (skin/subcutaneous), size of ulcer, location, infection, history of previous ulceration and lower extremity amputation were recorded [22]. According to the PEDIS classification, grades of infection were defined as: Grade 1: No symptoms or signs, Grade 2: Inflammation of skin/subcutaneous tissues only, Grade 3: Extensive erythema deeper (>2 cm) than skin/subcutaneous tissues and Grade 4: Systemic inflammatory response syndrome) [22]. Aside from imaging techniques, the standard probe to bone technique for diabetic foot ulcers was also used to diagnose osteomyelitis in these ulcers [23]. This technique is a quick, low cost and efficient screening test for early diagnosis of osteomyelitis in patients with diabetic foot ulcers [23]. The UT Diabetic Wound Classification System was used to classify the ulcers into a single validated grading system [24].

Foot ulcer duration was categorised into <1 week, 1 week to 3 months and >3 months [25]. If more than one ulcer was present, the primary ulcer was defined as the ulcer with the largest cross sectional area (cm^2^) [16, 26]. The size of an ulcer was determined by using a felt tip pen to trace the wound margins and transferring the wound tracing into the medical record. The wound dimensions were obtained by measuring the length and width using a ruler whilst the depth was measured from the deepest area of the ulcer using a sterile probe to calculate the volume (cm^3^) (length x width x depth) of the ulcer. Re-ulceration was the indicator used to define a previous foot ulcer that has re-ulcerated on the same location. History of a foot ulcer indicated previous ulceration on any location of either foot. UT Wound Classification of 0A and 0C are considered completely epithelialised [27]. A traumatic event was defined as an acute injury such as a footwear rub, blister or an episode of plantar pressure overload. Causative factors also listed were post surgery, “other” (including reulceration) and unknown.

### Statistical analysis

Descriptive statistics to characterise the study sample were generated using SPSS 22.0 (IBM SPSS Statistics for Windows, Armonk, NY, USA). Normality of data distribution was assessed using the Kolmogorov–Smirnov test with Lilliefors significance correction. Consequently continuous non-parametric data are presented as median and interquartile range (IQR, 25^th^ and 75^th^ quartiles). Continuous data such as age and postcode scores (for socioeconomic status) were compared using the Mann Whitney *U* test and proportions using the chi squared (χ^2^) test. All inferential tests were two tailed and statistical significant differences were considered at the *P* < 0.05 level.

## Results

### Patient demographics, risk factors and co-morbidities

Overall, data from 278 patients were initially extracted from the Westmead Hospital Foot Wound Clinic Registry. Of these, 202 (73 %) patients with a foot ulcer at their initial visit were analysed. The remaining 76 (27 %) cases were excluded because the patient either had diabetes or the foot ulcer was categorised as healed at their initial visits upon further checking.

Patient demographics and physical characteristics are shown in Table 1. The median age was 78 years (IQR: 64–87) and the male-to-female ratio approximated 1:1. Men (median 74 years, IQR: 61–85) were younger than women (median 82 years, IQR: 66–88; *P* = 0.013). Of the 110 patients with a foot ulcer and BMI data, 49 (39.1 %) were of normal weight and 17 (15.5 %) were underweight (BMI ≤ 25 kg/m^2^). The remaining 47 (45.5 %) patients were overweight (BMI 25.0–29.9 kg/m^2^). There were 92 patients without height and weight data; BMI for these patients, therefore, could not be calculated. A total of 79 (38.6 %) patients were born overseas and were 86.1 % English-speaking. The two most prevalent co-morbidities were hypertension (*n* = 110, 54.5 %) and hyperlipidaemia (*n* = 79, 39.1 %). Neuropathy was present in 34.7 % (*n* = 70) of patients. Over 50 % of patients with foot ulcers were smokers or ex-smokers. Thirty percent of patients had a history of a foot ulcer. The complete list of medical history and lifestyle risk factors are shown in Table 2.

**Table 1:** Demographics and physical characteristics of the sample (n = 202)

Characteristic	Total participants
Age (median years, IQR^‡^), *n* = 202	78 (64–87)
Gender, Male, no. (%), *n* = 202	109 (54.0)
Height (median metres, IQR^‡^), *n* = 125	1.7 (1.6–1.8)
Weight (median kg, IQR^‡^), *n* = 119	68 (55–84)
BMI (median kg/m^2^, IQR‡), *n* = 110	24 (20–28)
BMI category^a^, no. (%), *n* = 110	
Underweight	17 (15.5)
Normal	43 (39.1)
Overweight	37 (33.6)
Obese	11 (5.4)
Morbidly Obese	2 (1.8)
Socioeconomic^b^ median score(IQR^‡^), *n* = 202	996 (935–1037)
Nationality, no. (%), *n* = 202	
Australian born	124 (61.4)
Born overseas	79 (38.6)
Marital Status, no. (%), *n* = 202	
Married or De Facto	92 (45.5)
Widowed	56 (27.7)
Single	30 (14.9)
Other	24 (11.9)

^a^Underweight defined as BMI below 18.5 kg; Normal was defined as 18.5–24.9 kg; Overweight was defined as BMI 25.0–29.9 kg/m^2^; Obese was defined as BMI 30.0–39.9 kg/m^2^; Morbidly Obese was defined as BMI > 40.0 kg/m^2^

^b^Australia Bureau Statistics postcode score

^‡^ IQR: 25^th^ to 75^th^ percentile

**Table 2:** Medical history and lifestyle risk factors of the sample (*n* = 202)

Variables	Number of participants (%)
Neuropathy	70 (34.7)
Hypertension	110 (54.5)
Hyperlipidaemia	79 (39.1)
History of ulcer (Healed)	61 (30.2)
Retinopathy	10 (5.0)
History of amputation	32 (15.8)
Angina/Infarct	34 (16.8)
Nephropathy	9 (4.5)
Renal Failure	9 (4.5)
Claudication	22 (10.9)
Cerebrovascular Accident	26 (12.9)
Transient Ischaemic Attack	10 (5.0)
Smoking, *n* = 201	
Smoker	35 (17.4)
Ex smoker	72 (35.8)

The median socioeconomic index score was 996 (IQR: 935–1034) for Australia (mean index = 1000) [19]. A low socioeconomic index score indicates relatively greater disadvantage whereas a high score indicates a relative advantage [20]. Of the 47.5 % (*n* = 96) patients with a foot ulcer from relatively advantaged areas (IRSD score >1000), 29.2 % (*n* = 28) had a history of ulceration and 18.8 % (*n* = 18) had a history of amputation. Of the 52.5 % (*n* = 106) patients from relatively disadvantaged areas (IRSD score of <1000), 31.3 % (*n* = 33) had a history of ulceration and 13.2 % (*n* = 14) had a history of amputation. There was no significant difference in IRSD scores between those with a history of ulceration (*P* = 0.583) or amputation (*P* = 0.874).

### Foot ulcer characteristics

202 patients in total presented with foot ulcers. 198 (98 %) foot ulcers were recorded as new ulcers during the initial visit and 4 (2 %) were recorded as re-ulcerations. Of the 202 patients, 18 (9 %) had multiple ulcers. Primary ulcer characteristics and UT Wound Classifications are shown in Tables 3 and 4 respectively. The UT Wound Classification has been validated only for diabetic foot ulcers. The median cross-sectional area of the primary ulcer was 1.2 cm^2^ (IQR: 0.3–5.0 cm^2^) and volume was 0.4 cm^3^ (IQR: 0.1–1.2 cm^3^). Ulcer cross-sectional area was <1 cm^2^ in 18 (8.9 %) patients, between 1 and 5 cm^2^ in 62 (30.7 %) patients, and >5 cm^2^ in 105 (52 %) and 17 (8.4 %) patients had missing data. Over 30 % (*n* = 62) were located on the plantar surface and 27 % (*n* = 54) on the dorsum of the foot. Overall the forefoot and digits accounted for 69.5 % (*n* = 140) of ulcer locations. Ulcer duration at initial visit was < 1 week for one patient (0.6 %), 1 week to 3 months for 73.6 % (*n* = 120) of patients and >3 months in 25.8 % (*n* = 42) of patients. The greatest ulcer duration at initial visit was 300 weeks. Predominant UT wound categories consisted of 1A (37.9 %), 1B (15.4 %) and 3B (9.7 %) (Table 4). A total of 38 (19.4 %) foot ulcers were classified using UT Classification System as category 3A.

**Table 3:** Primary ulcer characteristics of the sample

Characteristics	Total participants
Anatomical Region, *n* = 200	
Hallux, no. (%)	39 (19.5)
Digits, no. (%)	49 (24.5)
Forefoot, no. (%)	52 (25.5)
Midfoot, no. (%)	23 (11.5)
Heel, no. (%)	38 (19.0)
Location, *n* = 200	
Plantar, no. (%)	62 (30.5)
Dorsal, no. (%)	54 (27.0)
Lateral, no. (%)	25 (12.5)
Medial, no. (%)	25 (12.5)
Apex, no. (%)	35 (17.5)
Side, *n* = 199	
Right, no. (%)	109 (54.8)
Left, no. (%)	91 (45.2)
Duration (weeks), median (IQR^‡^), *n* = 163	8 (4–24)
<1 week, no. (%)	1 (0.6)
1 week – 3 months (12 weeks), no. (%)	120 (73.6)
>3 months (12 weeks), no. (%)	42 (25.8)
Size	
Length (cm), median (IQR^‡^), *n* = 185	1.2 (0.6–2.3)
Width (cm), median (IQR^‡^), *n* = 185	1.0 (0.5–1.8)
Depth (cm), median (IQR^‡^), *n* = 182	0.2 (0.1–0.4)
Cross sectional area (cm^2^), median (IQR^‡^), *n* = 185	1.2 (0.3–5.0)
Volume (cm^3^), median (IQR^‡^), *n* = 184	0.4 (0.1–1.2)

^‡^ IQR: 25^th^ to 75^th^ percentile

**Table 4:** Primary ulcer grade/depth according to The University of Texas classification system for diabetic foot wounds [24]

	Grade/Depth *N* = 195				
		0	1	2	3
		Pre- or post- ulcerative lesion completely epithelialised	Superficial wound not involving tendon, capsule or bone	Wound penetrating to tendon or capsule	Wound penetrating to bone or joint
Stage/Comorbidities *N* = 195	A	*n* = None	*n* = 74(37.9 %)	*n* = 4 (2.1 %)	*n* = 2 (1.0 %)
	B With infection	*n* = None	*n* = 30 (15.4 %)	*n* = 2 (1.0 %)	*n* = 19 (9.7 %)
	C With ischaemia	*n* = None	*n* = 32 (16.4 %)	*n* = 4 (2.1 %)	*n* = 7 (3.6 %)
	D With infection and ischaemia	*n* = 1 (0.5 %)	*n* = 8 (4.1 %)	*n* = 2 (1.0 %)	*n* = 10 (5.1 %)

Almost one third (*n* = 70, 34.5 %) of all ulcers were infected and Grade 2 was the most prevalent (*n* = 44 (21.7 %) followed by Grade 3 (*n* = 24, 11.8 %) (Table 5). A total of 38 (18.8 %) out of 202 patients with a foot ulcer presented with osteomyelitis, and of these 28 (74 %) were positively diagnosed using the probe to bone technique with 5 (13 %) confirmed by imaging, 3 (8 %) by biopsy and 2 (5 %) were unknown. The causes of foot ulceration were: post surgery (*n* = 15, 7.4 %), traumatic event (*n* = 138, 68.3 %), other reulceration (*n* = 45, 22.3 %) and unknown (*n* = 4, 2 %).

**Table 5:** PEDIS classification grades of infection

Grades of infection	Total participants (*N* = 202)
Grade 1 No symptoms or signs	124 (61.1 %)
Grade 2 Inflammation of skin/subcutaneous tissues only	44 (21.7 %)
Grade 3 Extensive erythema deeper (>2 cm) than skin/subcutaneous tissues	24 (11.8 %)
Grade 4 Systemic inflammatory response syndrome	2 (1.0 %)
Missing data	9 (4.4 %)

At the initial visit, the two most commonly prescribed offloading modalities were the Darco Medical Surgical post-op shoe (*n* = 34, 16.8 %) and Sports/Orthopaedic shoes (*n* = 30, 14.9 %). One patient (0.5 %) was provided with an irremovable total contact cast (TCC) and one patient (0.5 %) with a removable TCC. All TCCs (irremovable and removable) were constructed with 3 M Softcast and Primacast according to our standardised protocol [28]. In 27.2 % (*n* =55) of patients other types of offloading modalities were applied which included air mattress for heel pressure off-loading, 12 mm cellular urethane combination innersole (Poron, Rogers Corp., Woodstock, CT, USA), Forefoot Wedge Shoe and Eggshell Foam Boot.

Ten patients (5 %) were referred for further vascular investigations to assess arterial flow and improve circulation. Of these, one was referred for endovascular surgery, four for duplex arterial ultrasound, two for diagnostic angiogram, one for diagnostic angiogram and endovascular surgery, one for duplex arterial ultrasound plus endovascular surgery and one for duplex arterial ultrasound plus diagnostic angiogram. The predominant UT Wound grades for these 10 patients were 1C (*n* = 4, 40 %) and 1D (*n* = 3, 30 %).

Three (1.5 %) patients required amputations (1 major and 2 minor) after their initial visit due to infection. Of these, two patients were from a relatively disadvantaged area (IRSD score of < 1000). Only one patient had peripheral neuropathy and two were current smokers. There were no deaths during the period of study.

## Discussion

This is the first study to report the characteristics of non-diabetic foot ulcers from the large Australian catchment of Western Sydney. This may also be the largest study in Australia to date investigating the classification, characteristics and location of non-diabetic foot ulcers. Of the 202 patients with non-diabetic foot ulcers investigated in this study 54 % (*n* = 109) were male and 46 % (*n* = 93) were female. This is in contrast to the previous study of 195 patients with diabetic foot ulcers in Western Sydney which reported 66.2 % (*n* = 129) male predominance [15].

Apart from diabetes, a number of other disorders increase the risk of developing foot ulcers, such as PAD and peripheral neuropathy [4]. Over half of patients in this study were smokers or ex-smokers, which is a strong risk factor for PAD [29]. PAD is rarely the cause of foot ulceration, but is a contributing factor in poor or delayed healing of foot ulcers [30]. One third of patients (35 %, *n* = 70) had neuropathy as one of the co-morbidities, which is a known risk factor for patients with diabetes [4]. Other disorders contributing to ulcer development include end-stage renal failure, vitamin B12 deficiency, gout, rheumatoid arthritis, scleroderma and cerebral palsy, or any other condition that affects the circulation, structure or sensation of the feet [4]. Co-morbidities such as retinopathy, nephropathy and renal failure were also recorded in 4–5 % of our sample suggesting some may have had subclinical; or undiagnosed diabetes [31]. However, a laboratory blood and urine tests are required to confirm diagnosis of diabetes. Foot deformity (such as claw or hammer toes and hallux valgus) can also occur as a consequence of wearing poor or ill-fitting footwear or as part of a disease process such as diabetes or rheumatoid arthritis [4]. Foot deformity may result in increased foot pressures and risk of developing foot ulcers [32].

The median age of our sample was 78 years, which is statistically significantly higher than the median age of 67 years reported in patients with diabetic foot ulcers in Western Sydney [15]. These data are consistent with the study by Bristow [12] indicating that non-diabetic foot ulcers are more likely to affect those who are aged over 70 years.

This study suggested that BMI is also a factor differentiating diabetic and non-diabetic foot ulcers. Of patients with non-diabetic foot ulcers, fewer than half (*n* = 50, 40.8 %) the patients with non-diabetic foot ulcers in this study were overweight or obese compared to more than 70 % (*n* = 94) in those with diabetic foot ulcers [15]. Indeed, this study has shown that non-diabetic foot ulcers are more likely to occur in those who are underweight or normal weight. It is thought that that obesity is associated with diabetes [33].

Low socioeconomic status has been thought to contribute to the development of diabetic foot problems [34]. This is the first study exploring socioeconomic index scores of ambulatory Australian patients with non-diabetic foot ulcers. Westmead Hospital has a large catchment area and is culturally diverse with a variable socioeconomic mix [19]. According to the Postal Area (POA) spreadsheet for IRSD, a socioeconomic index score of 996 was identified in this non-diabetic sample [20]. This result is almost identical to the Westmead diabetic foot ulcer study (socioeconomic index score of 996) and suggests that socioeconomic status is not related to diabetic or non-diabetic foot ulcer in Western Sydney, Australia.

Forefoot and digital (including hallux) non-diabetic ulcers were present in 69.5 % (*n* = 140) of patients. This finding is similar to the 72.3 % (*n* = 141) reported in the diabetic population [15]. While diabetic foot ulcers are commonly located on the plantar aspect of the foot due to abnormal loading and the presence of neuropathy [35], in the current study, the plantar *and* dorsum of the foot were equally affected. The causes of ulcers were mainly because of trauma and possibly due to the presence of neuropathy. The prevalence of neuropathy was lower (*n* = 70, 35 %) in the non-diabetic cohort compared to (*n* = 141, 75.4 %) in those with diabetic foot ulcers [15]. There were 41 % (*n* = 25) of patients who had neuropathy with ulcers located on the plantar aspect of the foot as opposed to 31.5 % (*n* = 17) patients with ulcers on the dorsal aspect. The lower number of plantar ulcers were consistent with Bristow et al. [12] who reported only 12 % (*n* = 16) plantar surface compared to other (non-plantar) ulcer locations i.e. digits, heel and dorsum combined.

The ulcer types recorded were heterogeneous, ranging from superficial to deep involving tendon, bone and joint with infection and ischaemia based on the UT Wound Classification System. A total of 22.1 % (*n* = 43) patients had a UT Wound Grade 1C to 3C (with ischaemia) and 10.7 % (*n* = 21) Grade 0D to 3D (with infection and ischaemia). However, it should be highlighted that the UT Wound Classification has been validated only for diabetic foot ulcers. A total of 38.9 % (*n* = 70) of our cohort exhibited an infection which is lower than those in the diabetic population (*n* = 97, 49.7 %) [15]. People with diabetes are more prone to infections such as osteomyelitis which was confirmed by only 19.4 % (*n* = 38) with non-diabetic foot ulcers having osteomyelitis compared to 25.6 % (*n* = 50) patients reported in the diabetic foot ulcer study [15].

Although the probe to bone technique is a low cost and quick screening test, a bone biopsy is usually needed to confirm presence of osteomyelitis [23]. In addition, the probe to bone test has only been validated for detecting osteomyelitis in the diabetic foot [23, 36]. Other test such as imaging (e.g. computerised tomography scan, X-ray and magnetic resonance), can also be used to diagnose osteomyelitis.

The low utilisation rate of the provision of TCC’s at the initial visit is due to the fact that the patient must be scheduled an appointment to allow for sufficient time for application of the TCC, wound care and education. Furthermore, this also allows the patient to present to the appointment wearing suitable clothing and to organise transport to and from the hospital.

This study is not without limitation. First, the data reported were derived from a retrospective analysis of a single site and excluded other foot clinics in Western Sydney. However, it is also important to highlight that Westmead Hospital has one of the largest catchment areas in Australia taking into account the estimated resident population in Western Sydney of 876,500 in 2013 [37]. Secondly, while all patients were identified as non-diabetic, routine examination of blood glucose levels would have identified subclinical cases of diabetes and ensured a homogeneous sample. Thirdly, the University of Texas, PEDIS grades of infection and the probe to bone test require validation in patients with non-diabetic foot ulcers. Fourthly, duration of foot ulcer prior to initial visit was generally self-reported, which is subject to recall bias. Fifthly, the Foot Wound Clinic Registry Data Form has not been validated or assessed for inter-rater reliability and so interpretative errors relating to ulcer characteristics and classification may have occurred. However, to reduce the potential for error, the Foot Wound Clinic Registry includes training in all aspects of data collection and entry.

## Conclusion

There is a paucity of information on the characteristics of non-diabetic foot ulceration in the Australian health care setting. It is also important to acknowledge the considerably high number of patients without diabetes with foot ulcers attending the Foot Wound Clinic at Westmead Hospital. More valid and reliable clinical tools are required to measure specific high-risk factors or foot ulcerations within multiple at risk population. In contrast to diabetic foot ulcers, the study found that non-diabetic foot ulcers largely affect older males *and* females with normal BMI on the plantar *and* dorsal aspect of the foot with a duration of 1 week to 3 months. In accordance with diabetic foot ulcers, socioeconomic status was not related to non-diabetic foot ulcers in Western Sydney. However, based on our findings the epidemiological pattern of non-diabetic foot ulceration and its pathogenesis requires further investigation.

## Abbreviations

ABS: Australian Bureau of Statistics
BMI: body mass index
IQR: interquartile range
PAD: peripheral arterial disease
PEDIS: perfusion, extent/size, depth/tissue loss, infection and sensation
SA1: statistical area level 1
SEIFA: socio-economic indexes for areas
TCC: total contact cast
UT: University of Texas
